# Multiple Primary Malignancies in Patients With Hepatocellular Carcinoma: A Largest Series With 26-Year Follow-Up

**DOI:** 10.1097/MD.0000000000003491

**Published:** 2016-04-29

**Authors:** Wei Xu, Wenjun Liao, Penglei Ge, Jinjun Ren, Haifeng Xu, Huayu Yang, Xinting Sang, Xin Lu, Yilei Mao

**Affiliations:** From the Department of Liver Surgery, Peking Union Medical College Hospital, Chinese Academy of Medical Sciences and Peking Union Medical College, 1# Shuai-Fu-Yuan, Wang-Fu-Jing, Beijing, 100730, China.

## Abstract

Multiple primary malignancies (MPMs) are defined as 2 or more malignancies without subordinate relationship detected in different organs of an individual patient. Reports addressing MPM patients with hepatocellular carcinoma (HCC) are rare. We perform a 26-year follow-up study to investigate characteristics and prognosis of MPM patients associated with HCC due to the scarcity of relative researches.

We retrospectively analyzed records of 40 patients who were diagnosed with MPM including HCC at the Departments of Surgery at Peking Union Medical College Hospital during 1989 to 2010. Their clinical characteristics and postoperative survival were compared with those of 448 patients who had HCC only during the study period.

Among the 40 MPM patients, 11 were diagnosed synchronously and 29 metachronously. The most common extra-hepatic malignancies were lung cancer (15%), colorectal (12.5%), and thyroid carcinoma (12.5%). MPM patients had a negative hepatitis B virus infection rate (*P* = 0.013) and lower median alfa-fetoprotein (AFP) level (*P* = 0.001). Post-operative 1-, 3-, and 5-year overall survival (OS) rates for MPM patients were 82.5%, 64.5%, and 38.6% respectively, and showed no significant difference with those of HCC-only patients (84.7%, 54.2%, and 38.3% *P* = 0.726). During follow-up, 24 MPM patients died, including 17 (70.8%) who died of HCC-related causes. In univariate analysis, synchronous diagnosis, higher gamma glutamyltransferase (GGT) and/or AFP levels, tumor >5 cm and vascular invasion were significantly associated with shorter OS, but only tumor size was an independent OS factor in Cox modeling analysis.

HCC should be considered as a potential second primary for all cancer survivors. Most MPM patients died of HCC-related causes and showed no significant difference in OS compared with HCC-only patients. Tumor size of HCC, rather than MPMs itself, was the only independent OS predictor for the MPM patients.

## INTRODUCTION

Multiple primary malignancies (MPMs) were first described according to the 1932 definition of Warren and Gates: each tumor has to present definite attributes of malignancy, the tumors have to be histological distinctive and the possibility of one being a metastasis of the other must be ruled out.^1^ Thanks to continually improving screening programs, diagnostic, and treatment methods, survival rates for newly diagnosed cancer patients are increasing. This improvement has led to a steady increase in the number of newly diagnosed MPM patients.^2^ In the United States, MPMs constitute 18% of all cancers diagnosed; in European countries, such as the Czech Republic, the MPM incidence is more than 11%.^3^

Hepatocellular carcinoma (HCC) ranks fifth in cancer incidence and third in cancer mortality worldwide.^4^ Although less than 1% of MPM patients reported had HCC in 1990s,^5^ longer overall survival (OS) of oncology patients elevated the risk of MPM significantly. By 2002, liver cancer was frequently diagnosed with other major malignant tumors; it was found in 11.5% of all MPMs in Korea.^6^ MPM patients who develop HCC over a long-term follow-up are no longer considered unusual, and clinicians increasingly need to consider the development of multiple primary cancers with HCC.

Information regarding the MPM patients with HCC is important, as it could clarify etiological factors and may verify the need to screen for associated malignancies during patient follow-up. Understanding of clinicopathological features and prognostic factors are also needed to facilitate appropriate management of MPM patients. However, knowledge of characteristic and outcomes of MPM patients remains limited.

To our knowledge, only studies with cohorts of 30 patients or fewer have been performed in Japan or Western countries for MPM patients with HCC who had received radical hepatectomy.^7,8^ The clinicopathologic characteristics and outcomes of MPM patients are poorly understood, especially in Asian countries. This retrospective study includes the largest sample size than any other researches and 26 years follow-up time, in order to characterize MPM patients and to explore their long-term prognosis.

## METHODS

Between January 1989 and September 2010, 40 patients with HCC that had been treated with radical hepatectomies were diagnosed with extra-hepatic primary malignancies at our institution; we regarded these patients as the target group (MPM group). Over the same period, 448 others with HCC only received hepatectomies; these patients were defined as the control group. In both groups, HCC was diagnosed on the basis of the histopathology from hepatectomy samples. The extra-hepatic primary malignancies were diagnosed on the basis of histopathology from resection (36/40) or biopsy (4/40) samples. A diagnosis of HCC as a second primary malignancy should be pathologically confirmed, as the liver is a common site for metastases and imaging findings may be atypical. To avoid the possibility of misdiagnosis between HCC and metastatic carcinoma, MPM patients who were diagnosed only by clinical methods were not included in this analysis. The MPM group was further classified into synchronous (2 malignancies diagnosed within a 6-month period) or metachronous (detected more than 6 months apart). The study protocol was approved by the Ethics Committee of Peking Union Medical College Hospital.

The preoperative data of patients’ clinical characteristics including age, sex, family history, serum hepatitis B virus (HBV), surface antigen (HBsAg), hepatitis C virus (HCV) antibody, serum alfa-fetoprotein (AFP) were collected, and histopathologic information regarding tumor number and size, tumor location, vascular invasion, nodal status, and cirrhotic change in background liver were recorded. Tumor differentiation was graded by the Edmondson grading system.^9^ The TNM staging system was used to assess HCC stage. Time for HCC surgeries, blood loss, and blood transfusion were recorded. Time for surgeries was defined as the time from the beginning of surgery to patients’ awakening from anesthesia. Median survival, and cumulative 3-year and 5-year survival rates were calculated. OS was defined as the interval between surgery and death or the last date of follow-up. Curative therapy for the extra-hepatic primary malignancies was defined as treatment with intent to cure, such as the surgeries for malignancies of breast, thyroid, digestive system, and respiratory system, or radial or chemical therapy for malignancies of blood system, while other treatment methods were regarded as palliative therapy.

Clinical and pathological factors were compared using either Fisher exact test or Pearson *χ*^2^-test, as appropriate. The survival rate was calculated using the Kaplan–Meier method. COX-regression analysis was performed to identify independent risk factors with hazard ratio (HR) and 95% confidence interval (CI). *P* < 0.05 was considered statistically significant. Data analysis was performed using SPSS 19.0 software.

## RESULTS

### Patient Characteristics

Of the 40 MPM patients, 11 were diagnosed synchronously, and 29 metachronously, with HCC; 18 patients’ extra-hepatic primary malignancies occurred prior to their HCC diagnoses (prior group), and 11 after their HCC diagnoses (post group). The most sites preceding or following HCC diagnoses were lung (6/40, 15%), colorectal (5/40, 12.5%), thyroid (5/40, 12.5%), breast (3/40, 7.5%), prostate (3/40, 7.5%), and sensory organs (3/40, 7.5%); 26 patients were treated by curative therapy and 4 by palliative therapy (Table 1).

**TABLE 1:**
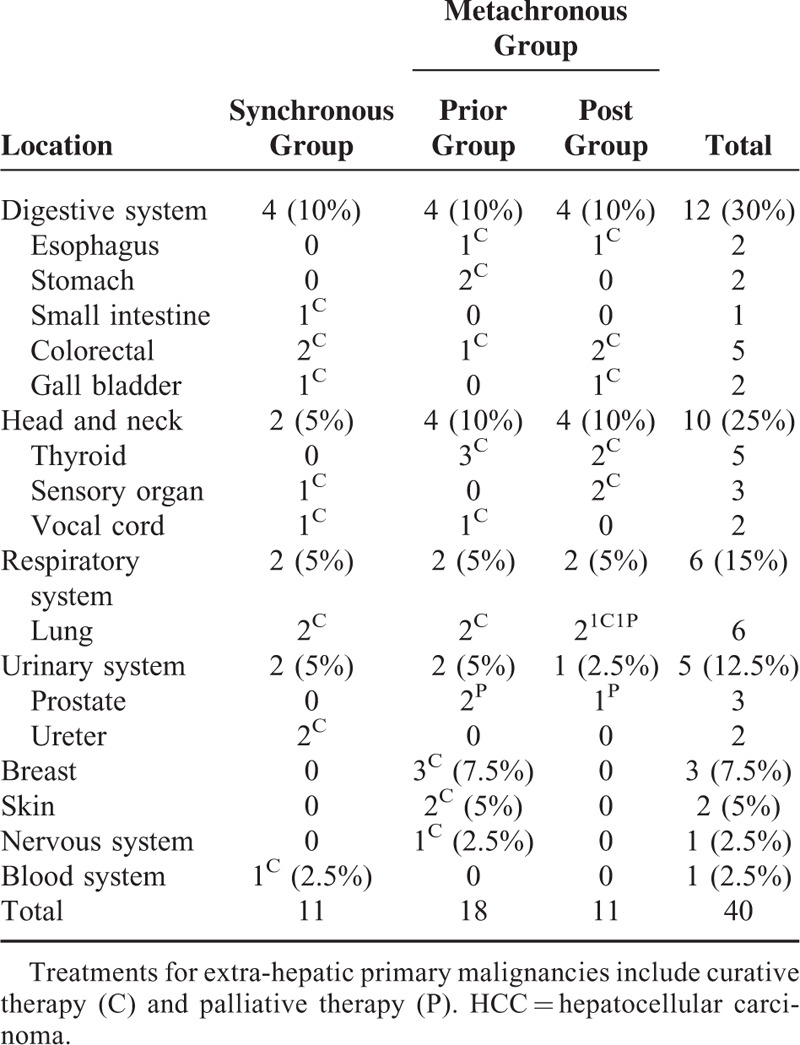
Site Distribution of Extra-Hepatic Primary Malignancies in Patients With HCC

**FIGURE 1:**
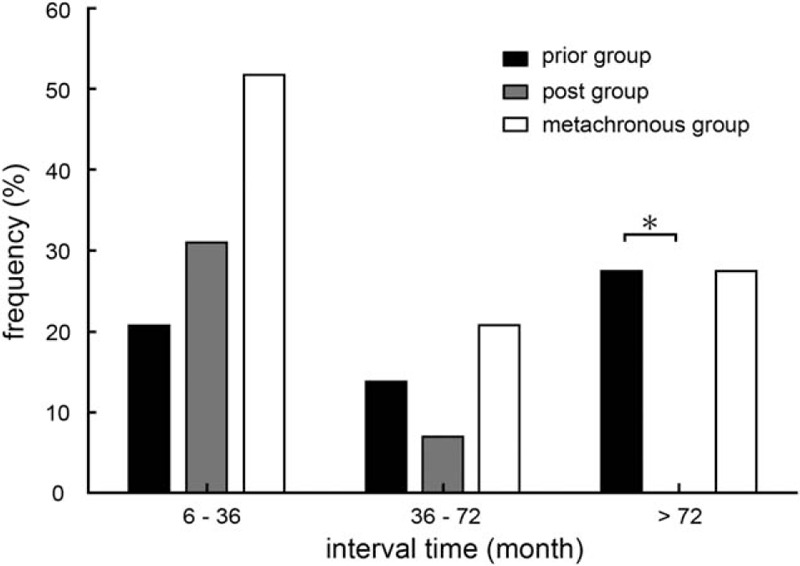
Diagnosis of secondary cancer by follow-up time after diagnosis of the first primary tumor, among patients whose first cancers were HCC (post), whose secondary cancers were HCC (prior), and those whose cancers were discovered more than 6 months apart (metachronous). ^∗^The post and prior groups differed significantly at interval time >72 months (*P* < 0.05). HCC = hepatocellular carcinoma.

Although diagnostic intervals between the 2 cancers ranged from 10 months to 21 years in the metachronous group (68.17 ± 73.99 months), 51.7% (15/29) of the metachronous patients were diagnosed with secondary cancers within 3 years of the initial cancer diagnosis (Figure 1). Moreover, 27.6% (8/29) of the MPM group were diagnosed after more than 6 years—all in the prior group, whose median interval time was significantly longer than that of the post group (93.89 ± 84.26 months vs. 26.09 ± 10.98 months, *P* = 0.003).

The MPM group included 36 men and 4 women. We detected HBsAg in 57.5% (23/40) patients; HCV antibody was positive in 17.5% (7/40); cirrhosis was present in 62.5% (25/40). Interestingly, we found that the proportion of patients with larger tumors (diameter >5 cm) in the synchronous group was significantly higher than that in metachronous group (9/11 vs.12/29 *P* = 0.034; Table 2). No other significant differences were found between the synchronous and metachronous groups.

**TABLE 2:**
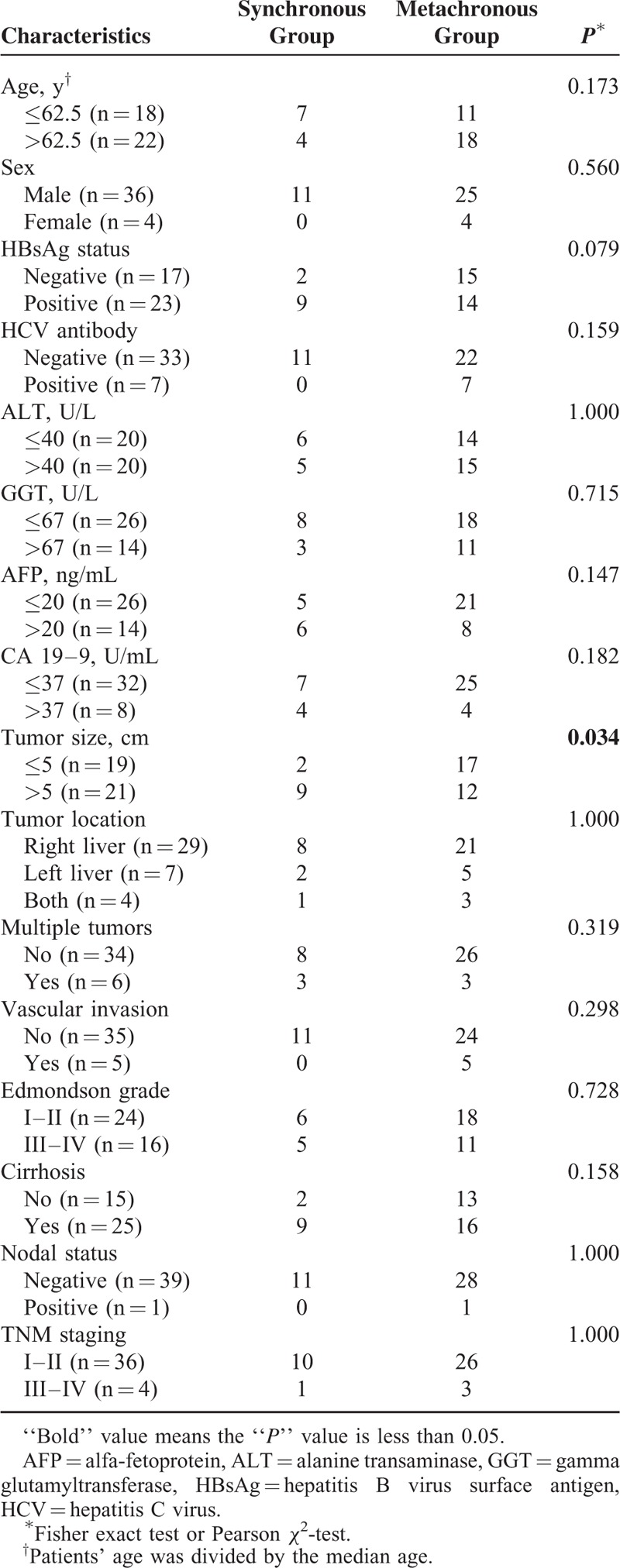
Comparison of Clinicopathological Characteristics Between Patients With Synchronous Group and Metachronous Diagnoses

Compared clinicopathological features between MPM patients and HCC patients in control group are shown in Table 3. The mean age of diagnosis in the MPM group was significantly older than that in the control group (62.58 ± 11.32 years vs. 55.69 ± 11.73 years, *P* < 0.001). Although more than half of MPM patients’ HBsAg statuses were positive (57.5%), the proportion of patients in positive HBsAg status in control group was significantly higher (76.3%) (*P* = 0.013). Further, more patients in control group showed abnormal serum AFP level (*P* = 0.001). However, no pathological features showed significant differences between the 2 groups.

**TABLE 3:**
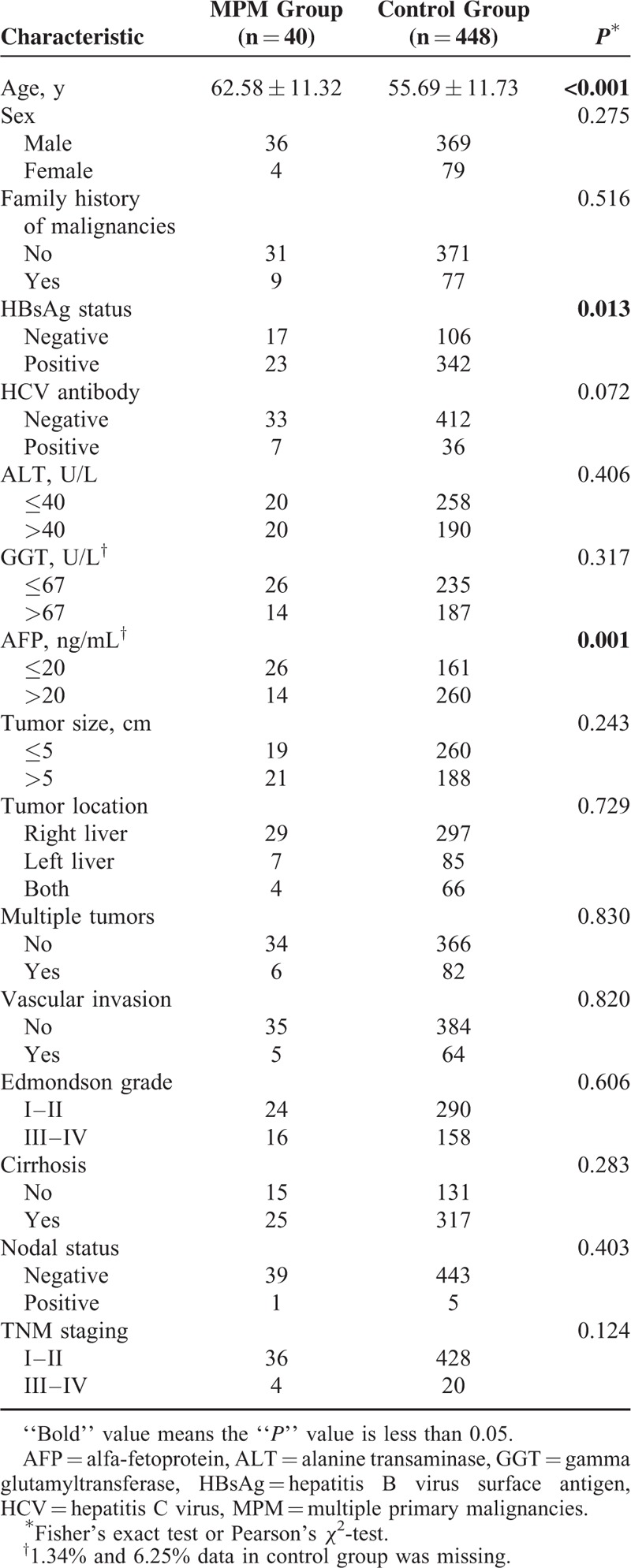
Compared Clinicopathological Characteristics Between MPM Group and Control Group

### Surgical Procedures

All MPM patients underwent surgeries for HCC including radical liver resections, as bi- or double segmentomies (n = 19), single segmentomies (n = 7), left lateral sectorectomies (n = 4), right anterior sector-plus segmentomies (n = 3), right anterior sectorectomies (n = 2), right hepatectomies (n = 2), right posterior sectorectomy (n = 1), left hepatectomy (n = 1), and left hepatectomy plus segmentomy (n = 1). Simultaneously, 4 patients underwent removal of portal vein tumor thrombi, 4 had extra-hepatic primary malignancies resected; 2 underwent cardiac peripheral vascular disconnections; and 3 received lymph node dissections because of enlarged nodes in the hepatoduodenal ligament region, including 2 found by intraoperative exploration and 1 whose suspected lymph node metastasis was diagnosed by preoperative magnetic resonance imaging (MRI). In 17 patients, we used Pringle's maneuver for intermittent hepatic inflow occlusion during surgery. Median surgery time was 180 min (range: 100–420 min) and median blood loss was 225 mL (range: 100–2000 mL). No patients died in the perioperative period.

Surgeries for the HCC-only patients included radical liver resections as bi- or double segmentomies (n = 143), single segmentomies (n = 93), right anterior sectorectomies (n = 59), right posterior sectorectomies (n = 42), left lateral sectorectomies (n = 25), right hepatectomies (n = 24), left hepatectomies (n = 24), right anterior sector-plus segmentomies (n = 16), left half liver sector-plus segmentomies (n = 9), right half liver sector-plus segmentomies (n = 8), and right half liver plus left lateral sectorectomies (n = 5). Fourteen patients underwent removal of portal vein or inferior vena cava tumor thrombus, 6 patients underwent splenectomy and cardiac peripheral vascular disconnection, 2 patients underwent phemister surgery simultaneously, and 200 patients underwent inflow vascular occlusion using Pringle's maneuver as mentioned above. Median time for surgery was 200 min (range: 60–600 min) and median blood loss was 400 mL (range: 50–15,000 mL). Four patients died in the perioperative period. The 2 groups did not significantly differ in surgery time (*P* = 0.099) or blood loss (*P* = 0.130).

### Patient Prognosis

Median follow-up time after HCC surgeries was 41.5 months (range: 2 months to 8.2 years). During the follow-up, 13 (32.5%) patients were still alive, 17 (42.5%) patients died of HCC-related causes, 2 (5%) of extra-hepatic primary malignancies-related causes and 5 (12.5%) of unclear causes. Three (7.5%) patients were unconnected for various reasons. Post-operative 1-, 3-, and 5-year survival rates for the 40 MPM patients were 82.5%, 64.5%, and 38.6%, respectively.

The effects of clinicopathological characteristics on survival were evaluated. Synchronous diagnosis, higher levels of GGT and AFP, tumor diameter >5 cm, and vascular invasion were significantly associated with poorer OS in univariate analysis (Table 4), but in Cox-multivariate analysis, only tumor size remained an independent predictor of survival (Table 5).

**TABLE 4:**
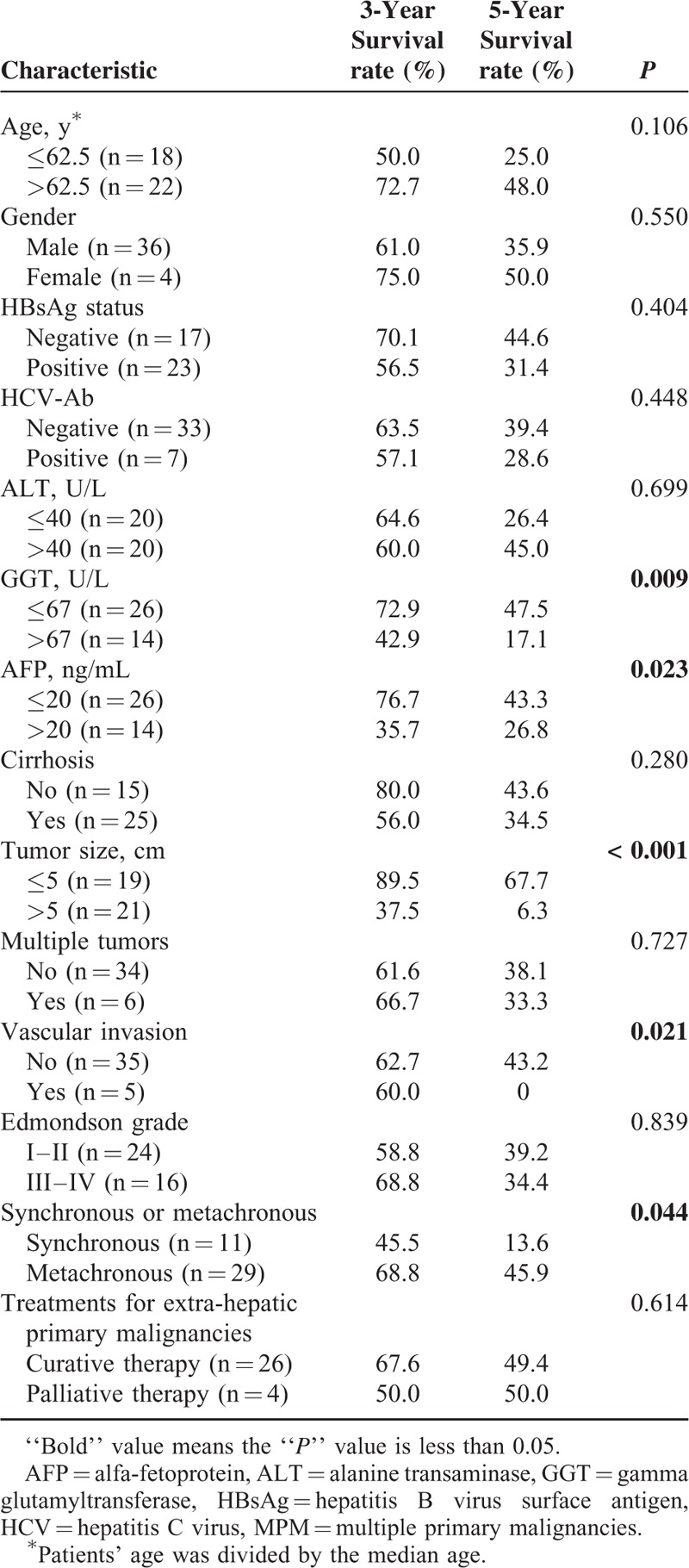
Univariate Analysis of Survival Risk Factors for MPM Patients

**TABLE 5:**
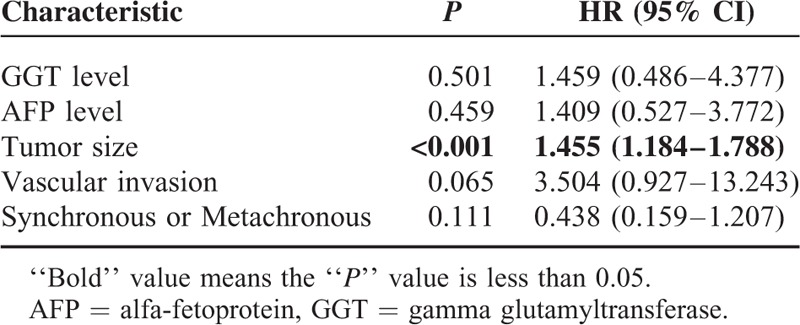
Cox Analysis of Survival Risk Factors for MPM Patients

**FIGURE 2:**
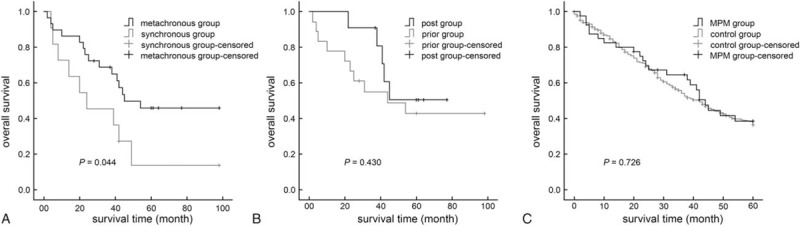
Comparisons of Kaplan–Meier curves between synchronous and metachronous groups (A); prior and post groups (B), and MPM and control groups (C). MPM = multiple primary malignancies.

The impact of second primary tumor on HCC survival was also estimated. Post-operative 1-, 3-, and 5-year survival rates for 448 HCC-only patients were 84.7%, 54.2%, and 38.3%, respectively, and did not significantly differ from those of the MPM group (*P* = 0.726, Figure 2C).

## DISCUSSION

Patients with malignancies have received increasing survival benefits from continuous progress in early cancer detection, diagnostic sub-classification, and targeted treatments. Along with increased life expectancy, cancer survivors are at higher risk of developing another malignancy compared with the general population. Reportedly, the prevalence of MPMs has increased, and 11.0% to 21.0% of all cancers have more than one primary in Western countries.^10^ The Surveillance, Epidemiology and End Results Program of the US National Cancer Institute estimated that 7.9% of cancer survivors were living with a history of more than 1 primary malignancy and MPMs now account for 16% of the newly diagnosed malignancies.^11^ Further, any survivor of cancer has twice the probability of developing a new second primary cancer than a cancer-free individual of the same age and sex.^12^ Thus, an increasing need exists to determine subsequent cancer risks, and to provide appropriate surveillance and management. Case reports or small-sized studies of MPMs that include HCC have been published in recent years,^13–15^ but information about their characteristics and outcomes is still limited, especially for those who underwent surgeries for HCC. In our series we had 40 MPM patients, the largest sample size ever, receiving radical resections for HCC and were diagnosed basis on their histopathology.

Although the etiology of HCC in MPM patients remains unclear, some evidence may be provided by their clinical features. HCC commonly arises in a background of chronic hepatitis and cirrhosis in Asian countries.^16^ In our study, 57.5% MPM patients had positive HBsAg statuses, which was significantly less than that the HCC-only control group (76.3%). HCV infection has also been suggested as a potential risk factor for HCC. Our study showed that 17.5% patients in MPM group were HCV^+^ and did not significantly differ from the HCC-only group. We are not surprised at the difference in HBsAg infection between the 2 groups, as reasons for HCC development in MPM patents may be more complex than those for the HCC-only group, although HBV and HCV infections were regarded as major causes for HCC.

MPM has been attributed to iatrogenic, environmental, and hereditary factors.^17^ Iatrogenic factors, such as anticancer treatments or radiation therapy, were considered as causes of MPM tumors. Reportedly, about 40% of patients with metachronous MPM had histories of receiving anticancer treatments or radiation therapy to attempt to cure their first cancers and consequently developed secondary tumors following their initial treatment.^18^ In our series, 37.9% (11/29) of MPM patients in the metachronous group had received chemotherapy or radiotherapy. Although we might have further considered the effects of radiation or chemical regimens, age at radiation exposure, and subsequent treatments, no clear differences were observed because of insufficient information.

Hereditary factors may be another cause of MPM tumors. Family history of malignancies, which is regarded as a risk factor for HCC, may also portend HCC development as a second malignancy. In our MPM group, 22.5% (9/40) had immediate family members with histories of cancer, which was similar to the patients in the HCC-only group (17.2% 77/371). We hypothesize that hereditary factors play a role in the process, but not solely in MPMs.

Aging is an important etiological factor in MPM patients. Using the Osaka Cancer Registry data, Tabuchi et al^19^ reported that 10-year cumulative risk of metachronous second primary cancer in Japanese male patients was 10.2% at 50 to 59 years of age, 16.2% at 60 to 69 years of age, and 21.8% at 70 to 79 years of age. In the present study, the mean age of HCC diagnosis in MPM patients was 62.58 ± 11.32 years, which was significantly older than that of the HCC-only control (55.69 ± 11.73 years). Furthermore, the mean ages of diagnosis did not significantly differ between the synchronous and metachronous groups (60.18 ± 8.86 vs. 63.48 ± 12.14 years, *P* = 0.418). This implies that older people have higher risks of developing second malignancies, without the choice for synchronous or metachronous.

Other risk factors such as BMI, immune status, and behavior change after the first primary malignancy may also contribute to HCC development in MPM patients,^20^ but more detailed investigation is needed. In most cases, inherited, iatrogenic, or viral factors are implicated; in other cases a clear etiopathogenesis is difficult to find, especially for synchronous MPMs. In our study, 2 synchronous HCC lesions without cirrhosis in background liver were surprisingly diagnosed by pathology after surgery for what were thought to be liver metastasis. One extra-hepatic synchronous tumor was unexpectedly found during the surgery, which was regarded as a benign lesion. Thus, the mechanism still needs further clarification.

Information about common sites of extra-hepatic malignancies may improve early detection in high-risk individuals.^21^ Gastric cancer has been reported as the most common extra-hepatic malignancy among MPM patients with HCC by Takayasu et al,^22^ along with colorectal cancer by Fernández-Ruiz et al,^23^ and nasopharynx cancer by Zeng et al.^24^ Unlike these previous findings, our study showed that the most common extra-hepatic malignancy was lung, followed by colorectal and thyroid. This circumstance may be partly attributable to different regions from which the study subjects were selected, for the most common forms of extra-hepatic malignancies were similar to the most common tumor types in China;^25–28^ and partly to the wide variation of multiple cancer distribution, which may occur as a result of random chance. Contrary to our expectation, screening for other possible malignancies in cancer survivors based on the most common sites is difficult because of the variable distribution of the extra-hepatic malignancy and any enrichment patterns can hardly have been proven by statistics yet. Establishment of a pair-wise association with HCC requires a more systematic and controlled approach.

Previous studies indicate that patients who initially presented with thyroid, urinary bladder, prostate, cervical, and uterine cancers were more liable to develop second malignancies, whereas those with hepatic cancers rarely developed a second malignancy. They hypothesized that this was, as HCC has a poor prognosis, HCC patients did not survive long enough to develop second primaries.^29^ HCC was among the four cancer sites with the lowest survival rates and consequently, the shortest duration of follow-up.^30^ However, about 40% (11/29) of our metachronously diagnosed patients were in the post group. The poor prognosis of HCC patients apparently does not affect the incidence of another primary tumor occurrence, and the possibility of developing extra-hepatic malignancies in HCC patients should not be ignored. Only the obviously longer interval time of the prior group can be explained partly by poor OS for HCC. Our MPM patients with interval times longer than 72 months were all in the prior group (Figure 1). In view of this pattern, physicians must consider the onset of HCC for each neoplasm, even many years after first diagnosis.

No consensus currently exists for a method of calculating the survival rate of MPM patients. Earlier researchers recommended basing the rate from the diagnosis of the final malignancy tumor, while others suggest calculating survival from the diagnosis of the first tumor, to account for the increased risk of malignancy during the first survival period.^31^ We focused on survival time after surgeries for HCC because most MPM patients died of HCC-related causes, which may indicate that MPM prognosis is largely determined by survival time after the HCC surgery. However, this may avoid the bias brought by longer intervals between MPM diagnoses, which could indicate a longer survival time.

Survival of MPM patients is reportedly similar to that of patients with single primary tumors.^23,32,33^ We had the same findings for post-surgical survival time (Figure 2C). Further, we found no significant difference in surgery-related parameters, such as surgery time and amount of bleeding, between MPM and control group. We speculate that a history of extra-hepatic tumor is not a direct obstacle to HCC resection. MPM itself does not necessarily indicate a poor prognosis, as long as adequate diagnosis and management are performed. However, HCC-related causes predominantly lead to MPM patients’ deaths; only 38.6% of patients in this study were still alive 5 years after their liver surgeries.

Male sex and old age have been shown by several studies to be risk factors for shorter survival in MPM.^34^ However, we found no statistical difference for OS in these terms. In the present study, serum GGT level, AFP level, tumor size, vascular invasion, and synchronous or metachronous diagnosis led to distinct outcomes. We verified these results with a Cox multivariate model, which only found tumor size, as a pathological feature, to be a significant independent risk factor for survival. This is an important new observation for patients who survived their first primary malignancy. Early detection and surgery for HCC would help improve OS in these patients.

Although the metachronous and synchronous groups significantly differed in OS (Figure 2A), metachronous or synchronous diagnoses were not independent OS factors in multivariate analysis. Metachronous malignant lesions were discovered because of careful follow-up of the first malignancy, during which extensive surveillance is carried out to locate possible metastases. This may explain why HCC lesions in metachronous group (mainly in the prior group) were found as smaller tumors than in the synchronous group, which may offer longer survival. Moreover, no significant difference in OS was found between the prior and post groups, which demonstrate that whether extra-hepatic malignancy was the initial or secondary malignancy did not influence OS after surgeries for HCC (Figure 2B). Another hypothesis is that as more time elapses between the 2 primary malignancies, the better the prognosis. Although the post group has a longer median period than the synchronous group before diagnosis of second malignancies, their OS rates did not significantly differ (*P* = 0.239). We found no relationship between second primary tumor development and MPM survival rate of MPM patients.

In summary, MPMs associated with HCC is rare. Our study provides the largest sample size of MPM patients ever, receiving radical resections for HCC. MPM patients were more likely to die of HCC-related causes even after receiving radical resection for HCC. Tumor size, rather than MPM itself, was the only independent predictive factor for OS in MPM patients. Follow-up for patients recovering from a first malignancy must be strictly observed, which could improve their chances for long-term survival. Because of the complex etiology and the variety of MPM cancer distributions, HCC should be considered as a potential second primary for every cancer survivors, even if not infected by HBV. Additionally, HCC patients, especially elderly ones, all malignancies must be considered risks of second tumor.

This study is subject to the limitations inherent in retrospective work with observation data collected at the specific point. It also represents the experience of a single tertiary referral center, and might not be generalized. The etiology of MPMs remains unclear, because risk factors known to be important to etiology, such as the details of chemotherapy or radiation therapy, could hardly be estimated in this study. Limitations of our study also include the confined sample size, although we have the largest sample size. A larger, multi-center study of patients from a multi-geographic patient base would be more conclusive.
